# Molecular signatures of alveolar type II cell differentiation in acute respiratory distress syndrome

**DOI:** 10.3389/fmed.2025.1649108

**Published:** 2025-09-17

**Authors:** Yu Zhang, Yazhe Wang, Chunsheng Wen, Haixu Wang, Liping Wang

**Affiliations:** The Fifth Affiliated Hospital of Xinjiang Medical University, Xinjiang, China

**Keywords:** acute respiratory distress syndrome, type II alveolar epithelial cells, cell differentiation, trajectory analysis, marker genes

## Abstract

**Background:**

Acute respiratory distress syndrome (ARDS) is a life-threatening lung condition with high morbidity and mortality.

**Methods:**

We analyzed publicly available single-cell transcriptomic and microarray datasets from murine ARDS models to characterize AT2 cell differentiation trajectories following lipopolysaccharide (LPS)-induced injury. Computational mapping of cell “trajectories” revealed distinct gene expression signatures associated with divergent repair outcomes. These findings were validated in bronchoalveolar lavage fluid (BALF) samples from ARDS patients and in an LPS-induced AT2-fibroblast co-culture model. Gene expression changes were examined at both the RNA and protein levels, and pathway enrichment analysis was used to explore underlying mechanisms.

**Results:**

Trajectory analysis revealed two major differentiation branches of AT2 cells: one enriched for fibrotic programs (Igfbp6, Gstm1, Mgp, and Lgals1) and the other linked to epithelial repair (Tgm2, Anxa1, Ankrd1, and F3). Both branches exhibited distinct gene expression patterns in patient BALF, which was consistent with scRNA-seq findings. Functional enrichment highlighted the Wnt signaling pathway as a key regulator in the injury group, which was validated at protein levels *in vitro*. The co-culture models showed that prolonged LPS exposure induced AT2 cell apoptosis, fibroblast activation, and extracellular matrix protein upregulation.

**Conclusion:**

This study highlights the important role of AT2 cell differentiation in shaping disease progression in ARDS and identifies potential molecular markers and signaling pathways involved in divergent repair outcomes. Our findings provide new insight into AT2 cell-driven lung repair and potential therapeutic targets.

## Introduction

Acute respiratory distress syndrome (ARDS) is a severe condition characterized by an acute onset of hypoxemia, bilateral pulmonary infiltrates, and diffuse alveolar damage, with high morbidity and mortality worldwide (1, 2).

The high mortality rate is largely attributable to the limited understanding of its molecular mechanisms and the lack of effective treatment strategies, making it essential to explore the underlying pathogenesis. The current management options, such as lung-protective ventilation strategies, prone position, and fluid-conservative treatment, have improved outcomes but have not substantially reduced overall morbidity or mortality (3, 4). Fibrosis and inflammatory responses are key pathological features of acute lung injury (ALI) (5). Typically, epithelial injury triggers the release of inflammatory mediators, facilitating neutrophil infiltration followed by macrophage recruitment to the injury site, which further amplifies cytokine production and modulates extracellular matrix components, including fibronectin, elastin, hyaluronic acid, and collagen (6).

When lung epithelial cells are damaged, a rare population of distal airway stem cells (DASCs) is activated to proliferate and migrate to the injured site, where they can differentiate into type I and type II alveolar epithelial cells (AT1 and AT2) (7). AT2 cells are widely recognized as a progenitor of the alveolar epithelium and play a pivotal role in lung repair and regeneration (8). The ARDS microenvironment contains multiple factors that influence epithelial cell differentiation, resulting in heterogeneity in cell differentiation status and cell fate (9, 10).

In recent years, single-cell RNA sequencing (scRNA-seq) has emerged as a powerful tool to characterize cellular states and transitions at single-cell resolution, enabling the comprehensive analysis of the transcriptomic landscape in disease tissues (11). For instance, scRNA-seq analyses have identified aberrant epithelial progenitor states in idiopathic pulmonary fibrosis (12), uncovered distinct macrophage phenotypes in ARDS (13), and mapped the temporal AT2 cells during regeneration following injury (14). By integrating single-cell genomics with trajectory analysis, researchers can classify cell subpopulations with distinct differentiation states. This strategy has been linked to the diagnosis, progression, and treatment outcomes of various diseases (15, 16). However, it remains unclear whether AT2 cells in ARDS exhibit distinct differentiation states and whether trajectory-based classification correlates with the biological behavior of ARDS.

Therefore, the present study specifically addressed the question of whether AT2 cells in ARDS exhibit distinct differentiation trajectories that correspond to different pathological processes, such as fibrosis or epithelial repair. With this objective, we integrated single-cell RNA sequencing and microarray datasets from mouse ARDS models to characterize the heterogeneity of AT2 cell differentiation, identify trajectory-specific marker genes, and elucidate their associated molecular pathways. Our goal was to uncover potential biomarkers and therapeutic targets that could improve the prediction of ARDS progression and inform personalized intervention strategies.

## Materials and methods

### Data collection

GSE132533 included scRNA-seq data from one control mouse and one mouse with 24-h injuries. Lung AT2 cells were isolated by flow cytometry and captured using the 10x platform. GSE106081 included gene expression profiling in lung AT2 from 10 lipopolysaccharide-induced lung injury and 5 control mice. These datasets were selected based on the following criteria: (1) inclusion of well-characterized ARDS models induced by clinically relevant agents, such as lipopolysaccharide (LPS), which closely mimic the inflammatory and fibrotic responses observed in human ARDS; (2) availability of transcriptomic data specific to AT2 cells, obtained either by scRNA-seq or microarray profiling; (3) presence of clearly defined control and injury groups, enabling robust differential expression analysis; and (4) sequencing depth and data quality to support reliable bioinformatic analyses.

### Processing of the scRNA-seq data of ARDS

The quality control (QC) of the scRNA-seq data from GSE132533 was performed using the Seurat R package (v4.0). Cells were retained if they met the following criteria: (1) expression of at least 200 genes, (2) <5% of total counts derived from mitochondrial genes, and (3) a total unique molecular identifier (UMI) count within three median absolute deviations (MADs) from the median of all cells to exclude potential doublets or low-quality cells. Data normalization was performed using the “LogNormalize” method in Seurat. Highly variable genes were identified using the “vst” method, and the top 2,000 genes were used for principal component analysis (PCA). Significant principal components (PCs) were selected with a significance threshold of a *p*-value of < 0.05.

Dimensionality reduction and clustering were performed using the t-distributed stochastic neighbor embedding (t-SNE) algorithm based on the selected PCs. Cell type annotation was conducted using the SingleR package with reference datasets from the Mouse Cell Atlas. Trajectory analysis is a computational method that arranges individual cells along a pseudotime axis according to similarities in their gene expression profiles, thereby inferring the dynamic progression of cellular states during biological processes such as differentiation. This approach enables the reconstruction of potential lineage relationships and the identification of branch points representing divergent cell fates. The Monocle R package was used for the analysis of single-cell trajectories.

### Differentially expressed genes

Differential expression analysis for GSE106081 was performed using the limma R package. The differentially expressed genes (DEGs) were screened based on |log2(FoldChange)| > 1, and a *p*-value of <0.05 when comparing ARDS and control.

### Enrichment analysis

Functional enrichment analysis for Gene Ontology (GO) and Kyoto Encyclopedia of Genes and Genomes (KEGG) pathways was performed using the clusterProfiler R package. A *p*-value of <0.05 was considered significantly enriched.

### Sample collection

Bronchoalveolar lavage fluid (BALF) samples were collected from five patients with ARDS and five healthy controls from the Fifth Affiliated Hospital of Xinjiang Medical University during diagnostic bronchoscopy, following standard clinical protocols. Baseline characteristics of the participating population are shown in Table 1. Sterile saline (20–30 mL) was instilled into a subsegment of the affected lung and gently aspirated. The recovered BALF was immediately placed on ice and centrifuged to pellet the cells. All subjects provided written informed consent. The study was in conformance with the guidelines of the 1975 Declaration of Helsinki and was approved by the ethics committee of the Fifth Affiliated Hospital of Xinjiang Medical University (No. XYDWFYLSk-2022-01).

**Table 1:** Baseline characteristics of ARDS patients and healthy controls.

Characteristic	ARDS patients (*n* = 5)	Healthy controls (*n* = 5)
Age (years), mean ± SD	58.4 ± 9.1	56.2 ± 8.7
Sex, male/female	3/2	3/2
PaO₂/FiO₂ ratio (mmHg), mean ± SD	142.6 ± 18.4	–
Underlying cause, *n* (%)		
Pneumonia	3 (60.0)	–
Sepsis	1 (20.0)	–
Aspiration	1 (20.0)	–
Comorbidities, *n* (%)		
Hypertension	2 (40.0)	–
Diabetes mellitus	1 (20.0)	–
COPD	1 (20.0)	–
Pre-sampling treatments, *n* (%)		
Mechanical ventilation	4 (80.0)	–
Corticosteroid therapy	2 (40.0)	–

### Cell model of ARDS treated with LPS

Human AT2 cells (A549, ATCC^®^ CCL-185™) and human lung fibroblasts (MRC-5, ATCC^®^ CCL-171™) were obtained from the American Type Culture Collection (ATCC) and maintained in DMEM (Thermo Fisher Scientific, California, USA) supplemented with 10% fetal bovine serum (Thermo Fisher Scientific, California, USA) and 1% penicillin–streptomycin (Thermo Fisher Scientific, California, USA) at 37 °C in 5% CO_2_. AT2 cells were seeded in the lower chambers of a Transwell system (0.4-μm pore size), while MRC-5 cells were cultured in the upper inserts for indirect co-culture. Four experimental groups were established: AT2, AT2+LPS, AT2+MRC-5, AT2+LPS+MRC-5. LPS treatment was performed at 1 μg/mL, with fresh LPS-containing medium replaced every 24 h for 5 consecutive days in the fibrosis-like model.

### Quantitative real-time polymerase chain reaction (qRT-PCR)

Total RNA was isolated from BALF samples and cells using the TRIzol reagent. Reverse transcription for mRNA and miRNA expression was performed using PrimeScript™ RT Master Mix (TaKaRa, Dalian, China). The qRT-PCR was carried out using the SYBR Green Master Mix (Invitrogen, California, USA) according to the manufacturer’s instructions. The primer sequence of genes is shown in Supplementary Table S1. The relative expression of genes was calculated using the 2^–ΔΔCT^ method. Genes were normalized to glyceraldehyde 3-phosphate dehydrogenase (GAPDH).

### Western blotting

For protein analysis, BALF samples and cells were lysed in RIPA buffer (Beyotime, China) containing 1% protease inhibitor cocktail (Sigma-Aldrich, USA) and then centrifuged at 12,000×*g* for 15 min at 4 °C to remove debris. Protein concentrations were determined by bicinchoninic acid (BCA) assay (Beyotime). Equal amounts of protein (30 μg) were separated by 10% SDS-PAGE and transferred onto PVDF membranes (Millipore, USA). Membranes were blocked with 5% non-fat milk in TBST for 1 h at room temperature and then incubated overnight at 4 °C with primary antibodies (1:1,000, Abcam, UK). After washing, the membranes were incubated with HRP-conjugated secondary antibodies (1:5,000, Proteintech, China) for 1 h at room temperature. Protein bands were visualized using an enhanced chemiluminescence (ECL) detection system (Bio-Rad, USA) and quantified with ImageJ software (NIH, USA).

### ELISA detection of secreted profibrotic factors

The culture supernatants from cells were collected at the endpoint, centrifuged at 1,000×*g* for 10 min to remove cell debris. The concentrations of connective tissue growth factor (CTGF), matrix metalloproteinase-2 (MMP2), transforming growth factor-beta (TGF-*β*), and tissue inhibitor of metalloproteinases-1 (TIMP-1) were determined using commercially available ELISA kits (Biosharp, China) according to the manufacturer’s instructions. The absorbance was measured at 450 nm using a microplate reader (BioTek, USA).

### Flow cytometric analysis of apoptosis

The apoptosis of AT2 cells was quantified using the Annexin V-FITC/PI Apoptosis Detection Kit (BD Biosciences, USA). Briefly, cells were harvested, washed twice with cold PBS, and resuspended in 1× binding buffer at a concentration of 1 × 10^6^ cells/mL. Then, 5 μL of Annexin V-FITC and 5 μL of propidium iodide (PI) were added to 100 μL of the cell suspension, gently mixed, and incubated for 15 min at room temperature in the dark. After adding 400 μL of 1× binding buffer, samples were immediately analyzed by flow cytometry using a BD FACSCanto II system (BD Biosciences, USA). Data were analyzed using FlowJo software (v10.8, BD Biosciences, USA) to determine the percentages of early and late apoptotic cells.

### Statistical analysis

Data analysis was performed using SPSS 20.0 software. Data were presented as mean ± standard deviations (SD). Student’s *t*-test was used to compare the differences between the two groups. A *p*-value of <0.05 was considered statistically significant. Test level *α* = 0.05 (two-sided).

## Results

### scRNA-seq reveals gene expression in AT2 cells of ARDS mice

**Figure 1:**
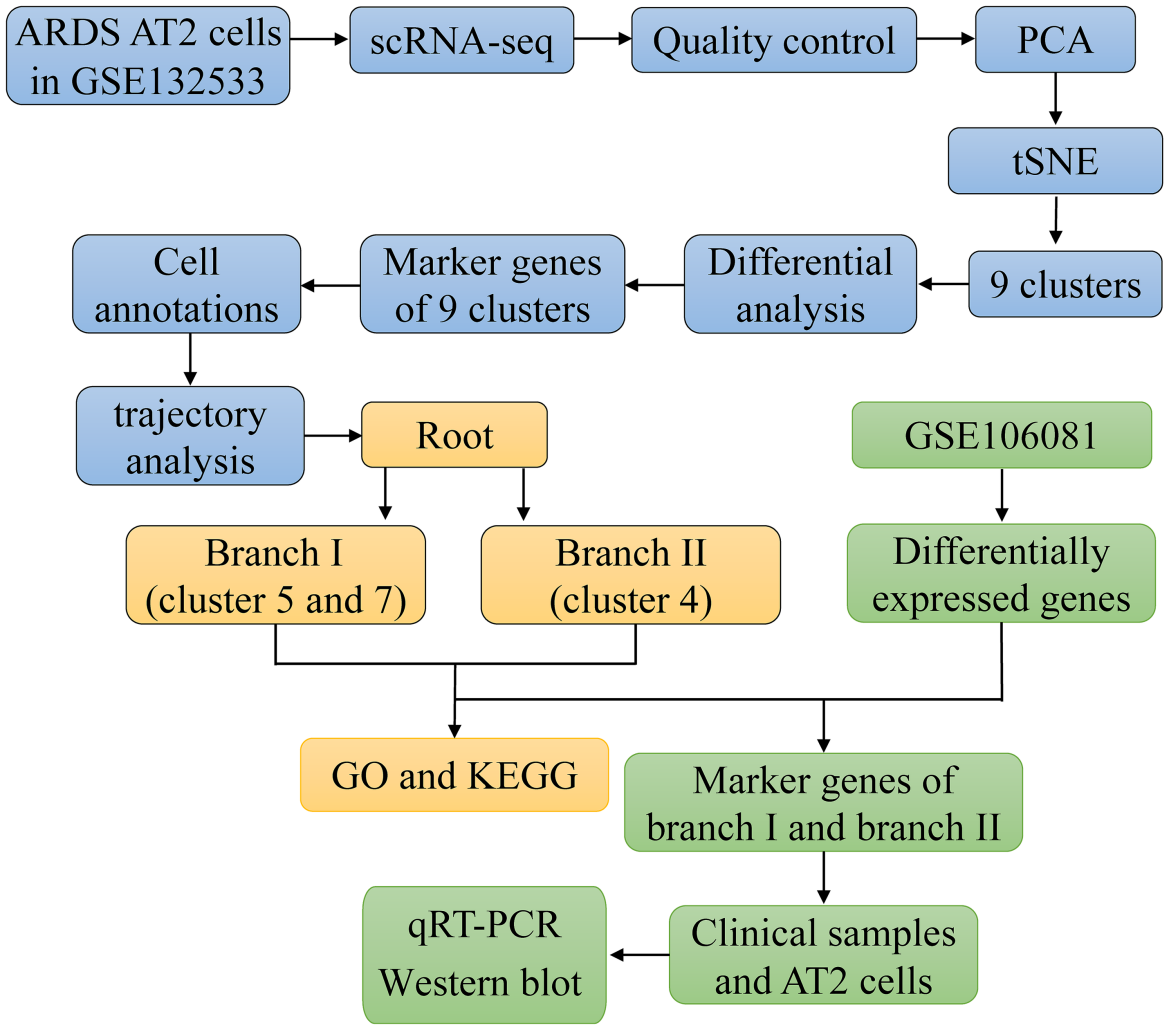
Flowchart of this study.

**Figure 2:**
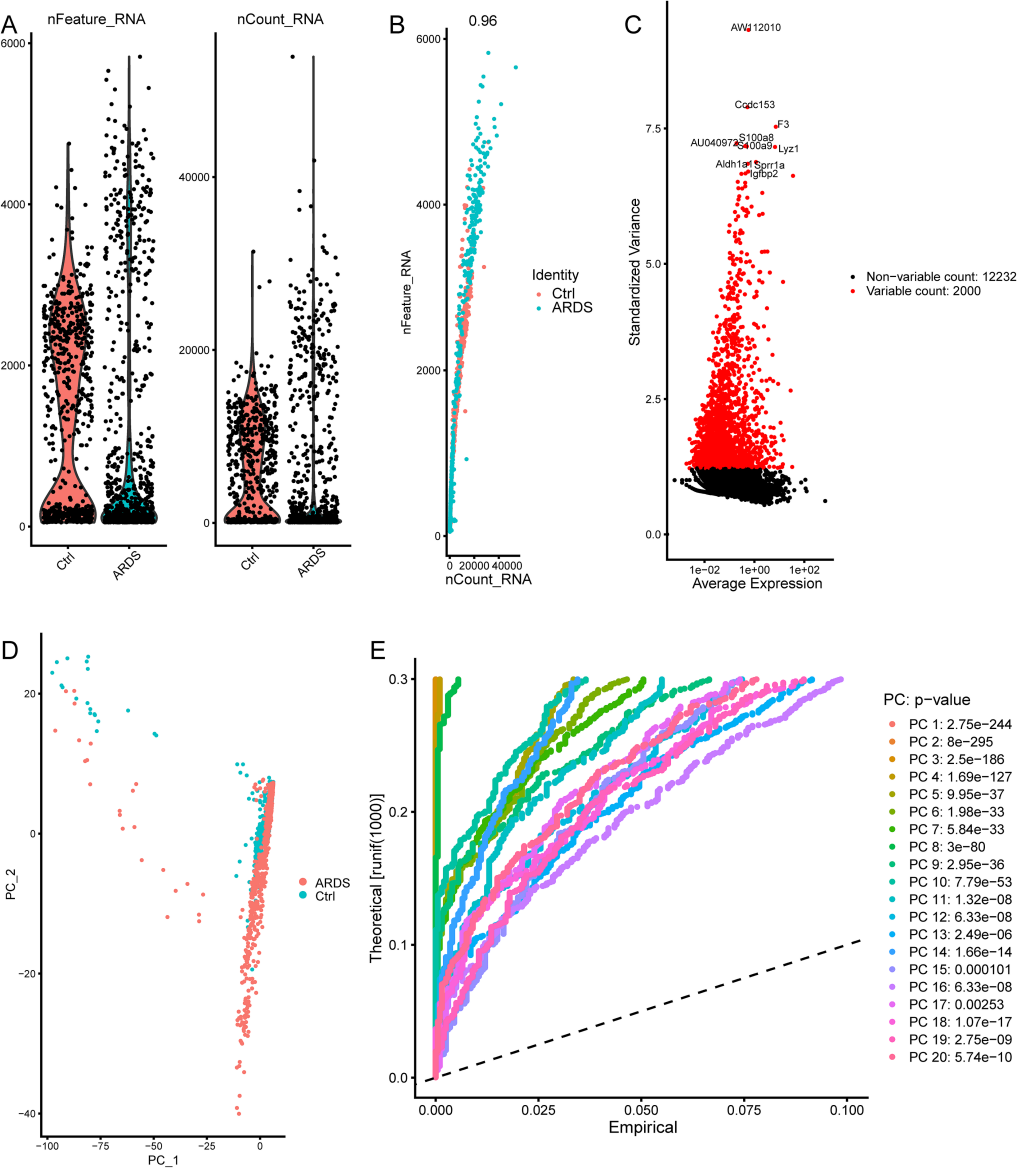
ARDS-related principal component genes were screened based on single-cell sequencing from the GSE132533 dataset. **(A)** Quality control of AT2 cells in ARDS. **(B)** The number of genes detected was significantly correlated with the depth of sequencing. **(C)** The variance diagram of gene expression. The red dots represent highly variable genes, and the black dots represent non-variable genes. **(D)** Principal component analysis (PCA) demonstrates no significant separations of cells between ARDS and control. **(E)** PCA identified 20 significant principal components (PCs).

The flowchart of the article is shown in Figure 1. To determine gene expression changes in AT2 cells, we analyzed the scRNA-seq of GSE132533. According to quality control and data standardization, a total of 1,586 cells were included in the analysis (Figure 2A). A significant correlation was observed between the number of genes detected and the depth of sequencing (Figure 2B). A total of 14,232 genes were included, and the variance analysis showed that 2,000 genes were highly variable in nature (Figure 2C). The PCA of 2,000 genes showed no significant separation of AT2 cells between ARDS patients and control patients (Figure 2D). By identifying the available dimensions and screening related genes by PCA, we finally selected 20 PCs for further analysis (Figure 2E).

### Heterogeneity of AT2 cells in ARDS mice

**Figure 3:**
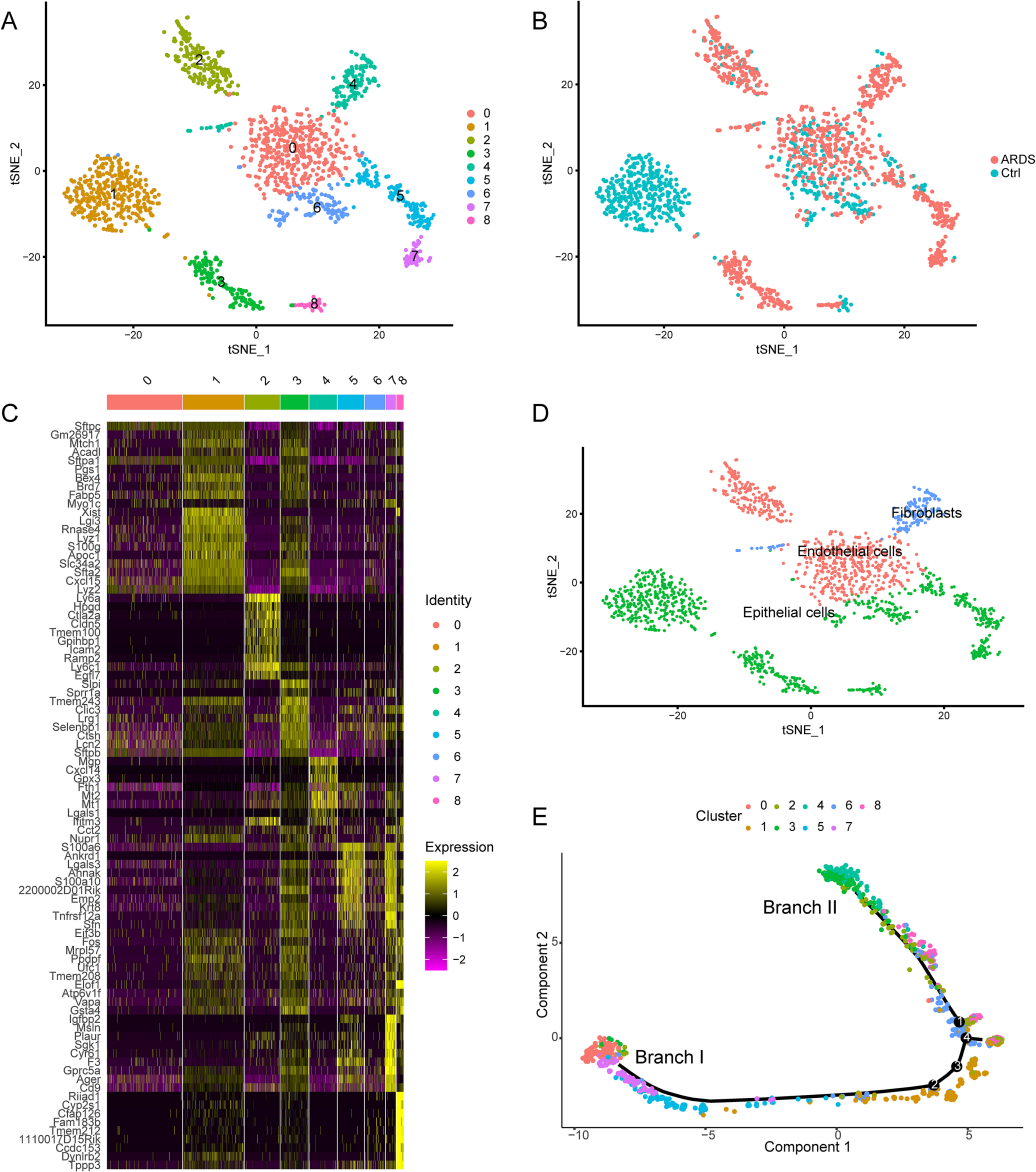
Identification of nine cell clusters with different annotations based on single-cell sequencing data from the GSE132533 dataset. **(A)** The tSNE algorithm reduced the dimensionality of 20 PCs and clustered them into 9 cell clusters. **(B)** The cell clusters were matched to the sample types. **(C)** The differential analysis identified 4,592 marker genes. The colors from purple to yellow indicate the gene expression levels from low to high. **(D)** All clusters of cells were annotated according to the composition of the marker genes. **(E)** Trajectory analysis revealed two subsets of AT2 cells with distinct differentiation patterns.

To determine the heterogeneity of AT2 cell differentiation during ARDS, we performed a cluster analysis of the cells. Through the *tSNE algorithm*, we clustered AT2 cells into 9 separate clusters (Figure 3A). Among them, cluster 1 matched the control group, and clusters 3, 4, 5, and 7 matched the ARDS group (Figure 3B). Afterward, differential expression analysis identified 4,592 marker genes in 9 clusters (Figure 3C). These clusters were annotated as three cell types according to the expression pattern of marker genes (Figure 3D). Clusters 0 and 2 were annotated as endothelial cells; cluster 4 was annotated as fibroblasts; and other clusters were annotated as epithelial cells. Through trajectory analysis, all AT2 cells were projected onto four roots and two branches, namely branches I and II (Figure 3E). Interestingly, all AT2 cells in branch I, defined here as type I AT2 cells, were from clusters 5 and 7, and the cells in branch II, defined here as type II AT2 cells, were all from cluster 4.

### Biological functions of the interested clusters

**Figure 4:**
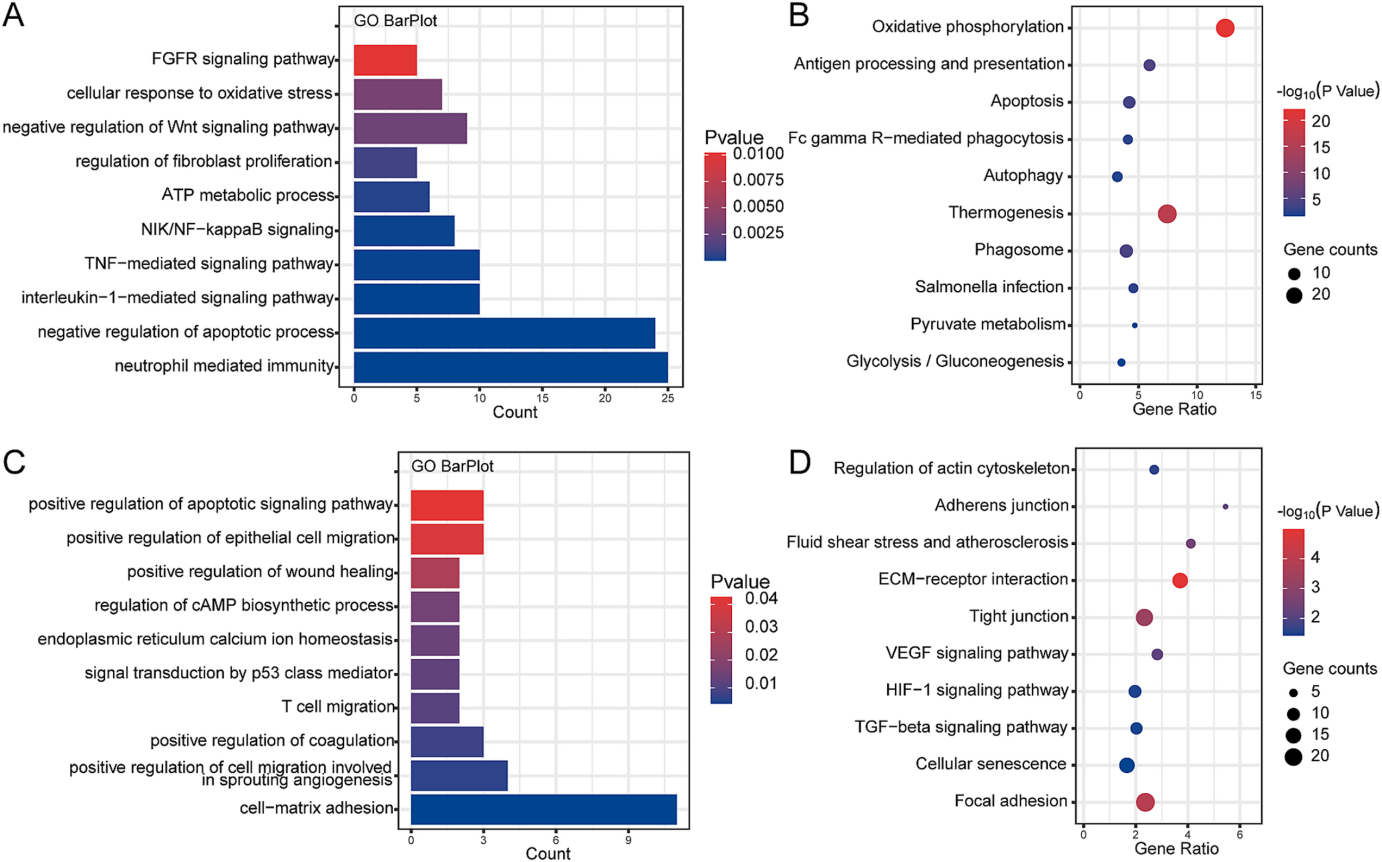
Enrichment analysis of interested clusters based on combined upregulated and downregulated genes within each cluster from the GSE132533 dataset. **(A)** The main biological processes of cluster 4 enrichment. **(B)** The main KEGG pathway of cluster 4 enrichment. **(C)** The main biological processes of cluster 5 and 7 enrichment. **(D)** The main KEGG pathway of cluster 5 and 7 enrichment.

We performed enrichment analysis on clusters of branches I and II, respectively, to explore potential damage mechanisms. The GO terms and KEGG pathways presented enrichment results derived from a combination of both upregulated and downregulated genes within each cluster. The results of enrichment analysis revealed that cluster 4 was significantly correlated with the negative regulation of the Wnt signaling pathway, neutrophil-mediated immunity, tumor necrosis factor-mediated signaling pathway, and regulation of fibroblast proliferation in GO (Figure 4A), as well as oxidative phosphorylation, apoptosis, autophagy, pyruvate metabolism, and glycolysis/gluconeogenesis in KEGG (Figure 4B). In addition, clusters 5 and 7 were significantly enriched in GO terms in the positive regulation of wound healing, of epithelial cell migration, of cell migration involved in sprouting angiogenesis, and of coagulation (Figure 4C), as well as KEGG pathways in adherens junction, VEGF signaling pathway, HIF-1 signaling pathway, and TGF-beta signaling pathway in Figure 4D. Therefore, branches I and II may represent two different heterogeneities of AT2 cells.

### Differential expression of branch marker genes

**Figure 5:**
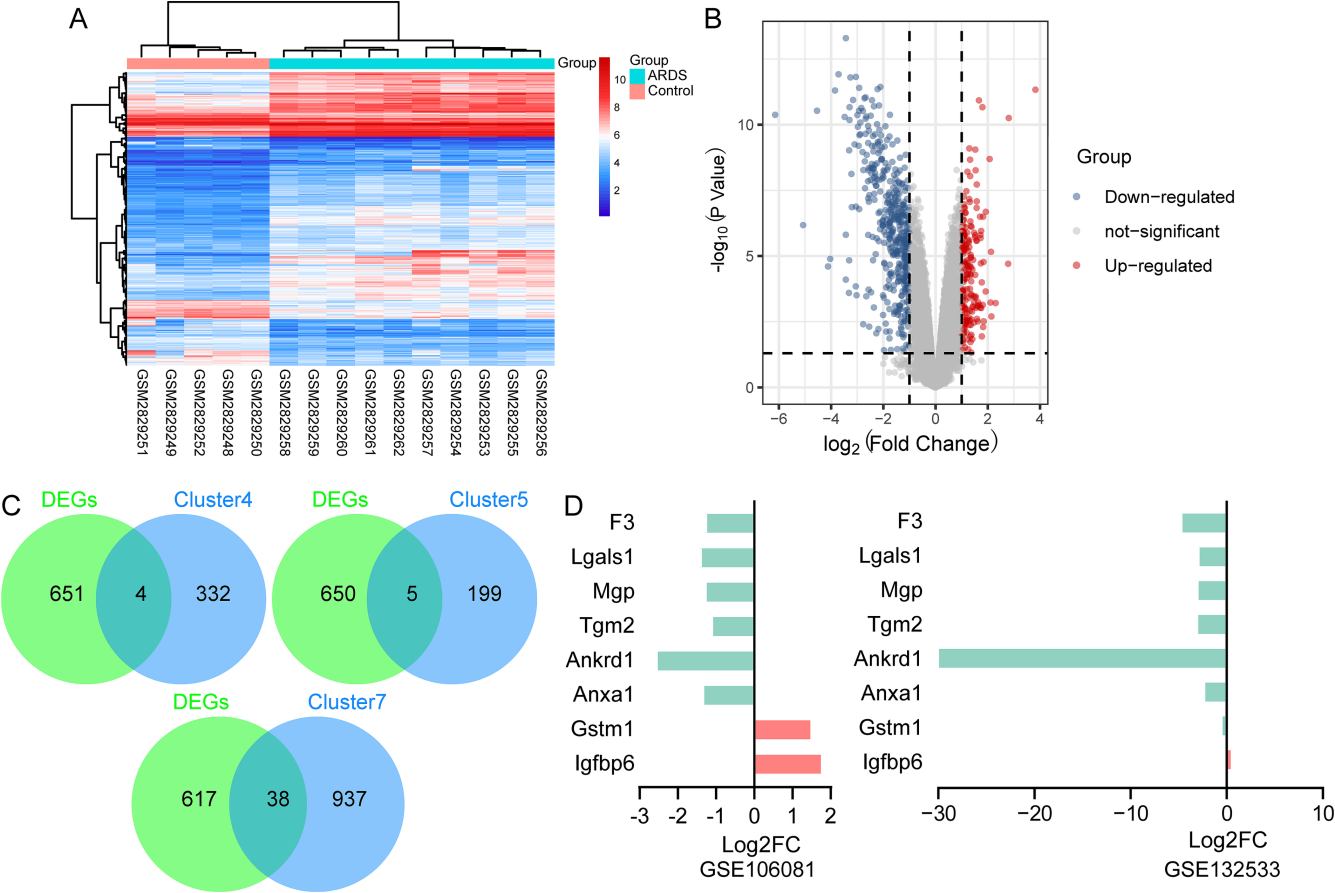
Identification of branch marker genes based on the GSE106081 dataset. **(A)** Heatmap of differentially expressed genes between ARDS and control in GSE106081 dataset. **(B)** Volcano map of differentially expressed genes between ARDS and control in GSE106081 dataset. **(C)** Intersection of differentially expressed genes with clusters. **(D)** Differential expression of key genes between ARDS and control in GSE106081 and GSE132533 datasets.

Then, we determined whether the observed AT2 cell subsets could be identified using array data. In the GSE106081 dataset, 655 DEGs were found between the ARDS and control groups (Figures 5A,B). Four of these DEGs belong to cluster 4: insulin-like growth factor binding protein 6 (Igfbp6), glutathione S-transferase mu 1 (Gstm1), matrix Gla protein (Mgp), and galectin-1 (Lgals1) were considered marker genes for branch II (Figure 5C). Five genes belonged to cluster 5, and 38 genes belonged to cluster 7 (Figure 5C). Therefore, Igfbp6, Gstm1, Mgp, and Lgals1 may be related to the fibrotic mechanism of ARDS, while Tgm2, Anxa1, Ankrd1, and F3 are expected to promote epithelial repair and regeneration. Among them, Tgm2 (transglutaminase 2), Anxa1 (annexin A1), Ankrd1 (ankyrin repeat domain 1), and F3 (coagulation factor III) existed in clusters 5 and 7 simultaneously and were considered marker genes of branch I. Compared with the control group, Gstm1 and Igfbp6 were upregulated in ARDS, while F3, Lgals1, Mgp, Tgm2, Ankrd1, and Anxa1 were downregulated (Figure 5D). Additionally, Igfbp6 was also upregulated in ARDS, and Gstm1, F3, Lgals1, Mgp, Tgm2, Ankrd1, and Anxa1 were downregulated in the GSE132533 dataset.

### Marker genes of the branch are associated with fibrosis in ARDS

**Figure 6:**
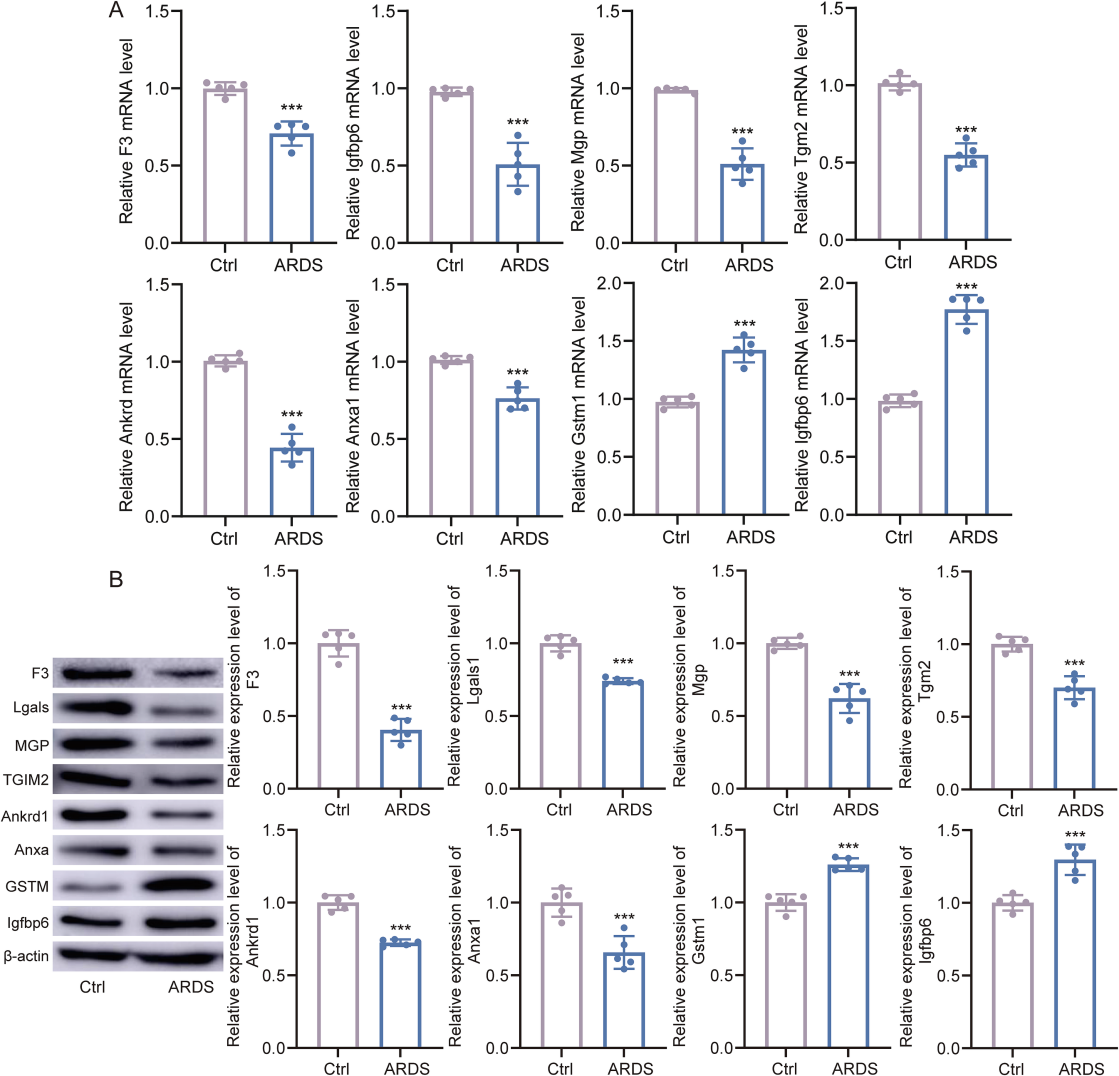
Marker gene expression in ARDS and controls. **(A)** The qTR-PCR experiments validated the differential expression of marker genes in bronchoalveolar lavage fluid (BALF) samples from ARDS patients and healthy controls. **(B)** Western blot analyses of marker genes in BALF samples from ARDS patients and healthy controls. *p < 0.05, **p < 0.01.

To verify the expression of marker genes in ARDS, we collected BALF from ARDS patients and healthy controls. The qRT-PCR results showed that, compared with controls, Gstm1 and Igfbp6 were significantly upregulated in ARDS patients, whereas F3, Lgals1, Mgp, Tgm2, Ankrd1, and Anxa1 were significantly downregulated (Figure 6A). Importantly, the trends in protein levels closely paralleled those observed at the mRNA level (Figure 6B), supporting the translational relevance of our transcriptomic findings.

### LPS-treated AT2/MRC-5 co-culture induces fibrosis-like remodeling

**Figure 7:**
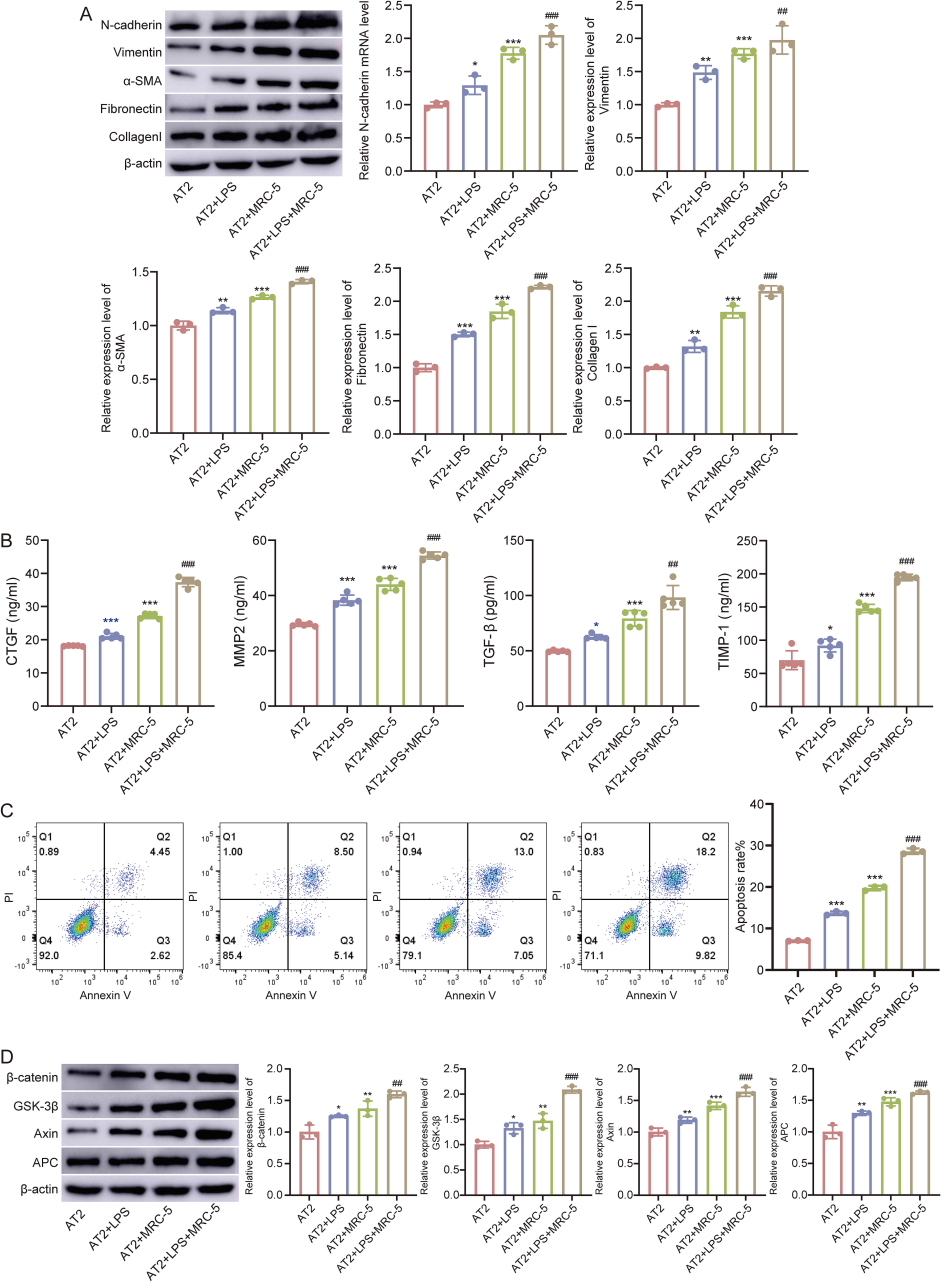
Epithelial injury drives fibrosis-like remodeling. **(A)** Western blot analysis of N-cadherin, vimentin, *α*-SMA, fibronectin, and collagen l across four groups (AT2, AT2+LPS, AT2+MRC-5, and AT2+LPS+MRC-5). **(B)** ELISA quantification of CTGF, MMP2, TGF-β, and TIMP-1 in culture supernatants. **(C)** Flow cytometric apoptosis (early/late) of AT2 cells. **(D)** Western blot analysis of β-catenin, GSK-3β, Axin, and APC. β-actin served as the internal control. Compared with AT2, **p < 0.01, ***p < 0.001, compared with AT2+LPS ###*p* < 0.001.

Fibrotic marker proteins are cooperatively upregulated by sustained LPS and co-culture. Western blotting analysis showed stepwise increases in classical fibrosis/EMT markers (N-cadherin, vimentin, *α*-smooth muscle actin (α-SMA), fibronectin, and Collagen I) in AT2+LPS compared with AT2, with further elevations in AT2+MRC-5 and the highest levels in AT2+LPS+MRC-5 after 5-day exposure (Figure 7A). ELISA of culture supernatants demonstrated significant increases in connective tissue growth factor (CTGF), matrix metalloproteinase-2 (MMP2), transforming growth factor-*β* (TGF-β), and tissue inhibitor of metalloproteinases-1 (TIMP-1) in LPS-treated groups relative to AT2 alone, with the greatest elevations in AT2+LPS+MRC-5 (Figure 7B). Flow cytometry (annexin V-FITC/PI) revealed higher fractions of early and late apoptotic AT2 cells after LPS, which further increased under co-culture conditions (AT2+LPS+MRC-5) (Figure 7C). These data indicate that epithelial injury and paracrine crosstalk with fibroblasts synergistically drive fibrosis-like remodeling.

To further elucidate the molecular mechanisms underlying these phenotypic changes, we focused on the Wnt signaling pathway, which was significantly enriched in the injury group based on our enrichment analysis. In the LPS-treated AT2/MRC-5 co-culture model, Western blotting analysis revealed marked upregulation of *β*-catenin, GSK-3β, Axin, and APC expression in AT2+LPS and AT2+LPS+MRC-5 groups compared with AT2, with the highest expression observed in the co-culture condition (Figure 7D). These molecular alterations are consistent with activation of the canonical Wnt/β-catenin pathway, suggesting that Wnt signaling may contribute to the fibrosis and the epithelial–mesenchymal crosstalk observed in the ALI/ARDS injury model.

## Discussion

The reprogramming of the alveolar epithelium is an early event in a multistep process that may eventually lead to end-stage fibrosis in IPF (17). In this study, we applied integrated single-cell RNA sequencing and microarray analyses to elucidate the differentiation characteristics and molecular programs of AT2 cells. Our results revealed that AT2 cells from ARDS lungs exhibit distinct transcriptional programs, with one trajectory enriched for epithelial repair markers and the other associated with fibrotic gene expression profiles. While the latter branch displayed upregulation of mesenchymal- and extracellular matrix-related genes, these changes likely reflect stress-induced phenotypic alterations or dysregulated repair responses within AT2 cells.

Using scRNA-seq technology, we found three AT2 clusters. Both endothelial cells and epithelial cells are associated with ARDS-related immune-inflammatory responses (18–20). An imbalanced inflammatory response, excessive accumulation, and activation of leukocytes and platelets in acute lung injury can cause increased permeability of alveolar endothelial cells and epithelial barriers (21). The processes involved in ARDS development include AT2 and fibroblast proliferation, as well as interstitial and alveolar fibrin accumulation (22). Mitigation of lung injury and pulmonary fibrosis in ARDS models is an important indicator of the therapeutic effect of drugs (23, 24).

Enrichment analysis of cluster 4 in branch II revealed significant enrichment in inflammatory response, autophagy, and fibroblast proliferation. Emerging evidence indicates that the aberrant activation of the Wnt signaling pathway plays a pivotal role in the development of fibrosis across multiple organs, including the liver, kidney, lung, heart, and skin (25). Among various proinflammatory cytokines, tumor necrosis factor-*α* (TNF-α) is a key mediator in the onset and development of ARDS (26). TNF-α enhances the expression of adhesion molecules in vascular endothelial cells, facilitates the activation and migration of fibroblasts and monocytes/macrophages, and amplifies inflammatory responses by inducing cytokine secretion (27). Additionally, studies have demonstrated that autophagy exerts a protective effect on ARDS induced by ischemia–reperfusion injury or viral infection (28, 29).

In addition, enrichment analysis of cluster(s) in branch I suggested that coagulation defects during ARDS exacerbate lung tissue injury by increasing alveolar capillary permeability, promoting alveolar hemorrhage, and intensifying airspace inflammation (30). The alveolar epithelium is a critical regulator of intra-alveolar coagulation (31, 32). Notably, activation of the coagulation system in ARDS is both a consequence and a driver of disease progression (33). The vascular endothelial growth factor (VEGF) family also plays a pivotal role in regulating vascular permeability and vasodilation, while supporting the stabilization of neovessel growth during wound healing (34). Hypoxia-inducible factor-1*α* (HIF-1α) acts downstream of TNF-α, modulates acute pulmonary inflammation, and controls alveolar capillary membrane permeability (35). Transforming growth factor-beta (TGF-*β*) is another major mediator in ARDS, contributing to increased epithelial and endothelial permeability and enhancing alveolar perfusion (36). Collectively, these findings highlight potential therapeutic targets for managing severe lung injury.

Among the marker genes of branch II, Igfbp6 shows transcriptional changes in acute lung injury (ALI) (37). It has been linked to fibrosis in experimental lung injury models and identified as a potential candidate gene for ALI (38, 39). Gstm1 plays a critical role in protecting cells from oxidative stress (40) and has also been reported as a candidate gene associated with ARDS susceptibility, based on its correlation with clinical course and patient outcomes (41). Mgp is highly expressed in pulmonary epithelial cells (42) and is essential for maintaining normal endothelial differentiation in progenitor cells (43). In Lgals1-deficient mice, circulating Th1 and Th17 cytokines levels are elevated, driving interleukin-(IL-)1β- and IL-18-mediated inflammation (44). Moreover, functional variants in LGALS1 that alter its expression have been associated with differential susceptibility to influenza A(H7N9) infection (45).

*In vitro* co-culture experiments reproduced the key transcriptomic patterns observed in the single-cell data. Specifically, fibrosis-associated proteins and profibrotic mediators were significantly increased in the LPS co-culture system, supporting the functional relevance of the single-cell findings.

Among the marker genes of branch I, Tgm2 is a multifunctional enzyme implicated in pulmonary inflammation and fibrosis (46). A significant decrease in Tgm2 expression has been identified as a target for altered Wnt signaling pathway activity during the AT2-to-AT1 differentiation process (47). AnxA1, an endogenous regulator of the anti-inflammatory process, exhibits potent anti-inflammatory effects in murine models of LPS-induced and ischemia–reperfusion-induced lung inflammation in mice (48, 49). Ankrd1 was upregulated in hyperoxia-exposed lung injury (50), and its overexpression has been shown to promote apoptosis (51). F3 interacts with downstream components of the coagulation cascade during vascular injury, exerting procoagulant effects (52). While F3-mediated fibrin formation is essential for hemostasis, wound repair, and tissue healing, excessive fibrin deposition in the lung is associated with the pathogenesis of ARDS and other pulmonary diseases (53).

Validation using BALF from ARDS patients confirmed the elevated expression of key branch II markers at both the RNA and protein levels. This cross-validation across species and sample types underscores the robustness of the identified signatures.

This study also has some limitations. First, all analyses were conducted using publicly available scRNA-seq and microarray datasets combined with *in vitro* validation, without the inclusion of *in vivo* animal models. Consequently, the cellular differentiation trajectories and molecular mechanisms inferred from our analysis require further confirmation in relevant ARDS animal models to account for the complexity of the lung microenvironment. Second, although BALF samples were used for clinical validation, the sample size was relatively small, which may limit the statistical power and generalizability of the findings. Third, the functional validation of branch-specific marker genes was limited to RNA and protein expression, and additional mechanistic experiments are warranted to establish causal relationships. Finally, although this study identified the Wnt signaling pathway as potentially involved in AT2 cell fate decisions, pathway activation was not comprehensively assessed *in vivo*, and thus, its role in ARDS progression remains to be fully elucidated.

## Conclusion

In summary, we delineated two distinct AT2 cell differentiation trajectories in ARDS, leading toward either epithelial repair or fibrosis. Key branch-specific markers were validated in patient BALF at both mRNA and protein levels, and functional modeling confirmed the involvement of the Wnt signaling pathway in fibrotic progression. These findings provide potential biomarkers and therapeutic targets for predicting and modulating ARDS outcomes.

## Data Availability

Publicly available datasets were analyzed in this study. This data can be found here: https://www.be-md.ncbi.nlm.nih.gov/geo/query/acc.cgi?acc=GSE132533; https://www.be-md.ncbi.nlm.nih.gov/geo/query/acc.cgi?acc=GSE106081.
