# Supplementary material for: Molecular signatures of alveolar type II cell differentiation in acute respiratory distress syndrome
Source: Front Med (Lausanne). 2025 Sep 17;12:1649108. doi: 10.3389/fmed.2025.1649108 (PMC12483913; doi:10.3389/fmed.2025.1649108)
Supplement: Supplementary file 1 [file Supplementary_file_1.docx]

Table S1. The primers of this study.

| Genes | Primers |
| --- | --- |
| *GAPDH* | F: 5′-TGTTGCCATCAATGACCCCTT-3′ |
|  | R: 5′-CTCCACGACGTACTCAGCG-3′ |
| *Igfbp6* | F: 5′-GACCAGGAAAGAATGTGAAAGTGA-3′ |
|  | R: 5′-GCTCTGCCAATTGACTTTCCTTAG-3′ |
| *Gstm1* | F: 5’-CTGCCCTACTTGATTGATGGG-3’ |
|  | R: 5’-CTGGATTGTAGCAGATCATGC-3’ |
| *Mgp* | F: 5′-CCATCTCTGCTGAGGGGATA-3′ |
|  | R: 5′-AACGCTTCACGAATTTGCGT-3′ |
| *Lgals1* | F: 5′-CTGTGCCTGCACTTCAACC-3′ |
|  | R: 5′-CAT CTGGCAGCTTGACGGT-3′ |
| *Tgm2* | F: 5′-ACTACAACTCGGCCCATGAC-3′ |
|  | R: 5′-TGGTCATCCACGACTCCAC-3′ |
| *Anxa1* | F: 5′-GCAGGAATATGTTCAAACTGTG-3′ |
|  | R: 5′-CCTTATGCAAGGCAGCGA-3′ |
| *Ankrd1* | F: 5′-AGCGCCCGAGATAAGTTGCT-3′ |
|  | R: 5′-CACCAGATCCATCGGCGTCT-3′ |
| *F3* | F: 5′-CAGACAGCCCGGTAGAGTGT-3′ |
|  | R: 5′-CCACAGCTCCAATGATGTAGAA-3′ |
